# Inhibiting Pyridoxal Kinase of *Entamoeba histolytica* Is Lethal for This Pathogen

**DOI:** 10.3389/fcimb.2021.660466

**Published:** 2021-04-16

**Authors:** Suneeta Devi, Priya Tomar, Khaja Faisal Tarique, Samudrala Gourinath

**Affiliations:** ^1^ Structural Biology Laboratory, School of Life Sciences, Jawaharlal Nehru University, New Delhi, India; ^2^ Department of Molecular Reproduction, Development and Genetics, Indian Institute of Science, Bangalore, India

**Keywords:** drug target, *Entamoeba histolytica*, enzyme kinetics, pyridoxal 5’-phosphate, pyridoxal kinase

## Abstract

Pyridoxal 5’-phosphate (PLP) functions as a cofactor for hundreds of different enzymes that are crucial to the survival of microorganisms. PLP-dependent enzymes have been extensively characterized and proposed as drug targets in *Entamoeba histolytica*. This pathogen is unable to synthesize vitamin B_6_ *_via_ de-novo* pathway and relies on the uptake of vitamin B_6_ vitamers from the host which are then phosphorylated by the enzyme pyridoxal kinase to produce PLP, the active form of vitamin B_6_. Previous studies from our lab shows that *Eh*PLK is essential for the survival and growth of this protozoan parasite and its active site differs significantly with respect to its human homologue making it a potential drug target. *In-silico* screening of *Eh*PLK against small molecule libraries were performed and top five ranked molecules were shortlisted on the basis of docking scores. These compounds dock into the PLP binding site of the enzyme such that binding of these compounds hinders the binding of substrate. Of these five compounds, two compounds showed inhibitory activity with IC_50_ values between 100-250 μM when tested *in-vitro*. The effect of these compounds proved to be extremely lethal for *Entamoeba* trophozoites in cultured cells as the growth was hampered by 91.5% and 89.5% when grown in the presence of these compounds over the period of 72 hours.

## Introduction

Amoebiasis is one of the most widespread and a global intestinal parasitic disease caused by *Entamoeba histolytica* (Walsh, 1986). It is a causative agent of amoebic dysentery leading to nearly 100,000 deaths and 35 to 50 million new infections per year across the globe (Li and Stanley, 1996; Petri et al., 2000; Stauffer and Ravdin, 2003; Bercu et al., 2007). The incidence of amoebiasis is higher in developing countries, particularly the tropics and subtropics, where amoebiasis is a major source of morbidity and mortality (WHO, 1998). PLP in *E. histolytica* is produced by *Eh*PLK by salvage pathway. PLP is the most active form of vitamin B_6_ (Gyorgy, 1956), which serves as a cofactor for various enzymes associated with racemization, transamination, decarboxylation and replacement reactions (Percudani and Peracchi, 2003). *E. histolytica* is microaerophilic in nature which can migrate from the intestines and invade other body organs to cause serious diseases. In particular, it may spread to the liver and brain to cause abscess and cerebral amoebiasis (Tanyuksel and Petri, 2003). Enzymes of the sulfur metabolism pathway such as O-acetylserine sulfhydrylase contain PLP as a cofactor and play an important role in the antioxidative defence mechanism (Raj et al., 2013; Jeelani and Nozaki, 2016) which has been targeted to develop inhibitors in *E. histolytica* (Nagpal et al., 2012; Ansari et al., 2016; Dharavath et al., 2020). Similarly, other PLP containing enzymes: aspartate aminotransferase, methionine gamma-lyase, cysteine desulfurylase, threonine dehydratase and phosphoserine amino transferase are equally important drug targets (Tokoro et al., 2003; Sato et al., 2008; Sato et al., 2017). We have been extensively characterizing some of these enzymes as potential therapeutic targets for structure based drug discovery, (Kumar et al., 2011; Kumari et al., 2019; Singh et al., 2019; Dharavath et al., 2020) and what remained something of utmost importance was to target pyridoxal kinase itself, which catalyses the formation PLP in *E. histolytica*. In our previous work, we have biochemically characterized and determined the crystal structures of pyridoxal kinase from *E. histolytica* where a single copy of this gene exists for the synthesis of PLP through the salvage pathway (Tarique et al., 2020). Notable structural differences from human PLK in the active site region of this enzyme were observed which means targeting this enzyme would be detrimental and affect the activity of all PLP-dependent enzymes of this human pathogen. In continuation of our previous work which showed *Eh*PLK to be necessary for survival and growth of *E. histolytica* (Tarique et al., 2020), we have gone further to identify two novel chemical compounds that inhibited the enzyme’s activity and severely affected the growth of this pathogen when tested in cultured cells.

## Materials and Methods

### *Eh*PLK Protein Expression and Purification

The expression of histidine-tagged *Eh*PLK in *Escherichia coli* and purification of the recombinant protein was done as described previously (Devi et al., 2019; Tarique et al., 2020). Briefly, transformed BL21(DE3) cells (Novagen) with *Eh*PLK construct were cultured in Luria Bertani broth (Hi-Media) for overnight at 37°C and later secondary culture was inoculated with one percent of the overnight grown cells. Culture was induced at OD_600_ = 0.7 to produce recombinant protein with 1 mM isopropyl-1-thio-β-D-galactopyranoside (G-Biosciences) and incubated for overnight at 18°C. The cell were harvested by centrifugation at speed of 7000 rpm and resuspended in lysis buffer. Cells were lysed using sonication and samples were run on high speed centrifugation (16000 rpm) at 4°C for 45 minutes. Recombinant protein present in the supernatant was then purified by immobilizing it onto Ni-NTA (GE Healthcare), followed by extensive washing with the buffer (30 mM Tris-HCl pH 8.0, 150 mM NaCl, 5 mM βME, 5% (v/v) glycerol) containing 20 mM of imidazole and eluting it with 300 mM imidazole in 30 mM Tris-HCl pH 8.0, 150 mM NaCl, 5 mM βME and 5% (v/v) glycerol. Purified fractions were analysed by 12% (w/v) SDS-PAGE for the presence of *Eh*PLK. *Eh*PLK containing fractions were pooled and concentrated using Amicon ultra centrifugal filters (Merck-Millipore). Gel-filtration chromatography was used for further purification. Concentrated protein was then loaded onto a 16/60 Superdex 200 column (GE Healthcare) pre-equilibrated with running buffer 30 mM Tris-HCl pH 8.0, 150 mM NaCl, 5 mM βME, 5% (v/v) glycerol.

### Screening of Inhibitors Against *Eh*PLK

Inhibitor screening against *Eh*PLK protein was carried out based on the *Eh*PLK·PLP complex structure, and PLP-binding site of the protein was defined as docking site for the inhibitor. Schrodinger’s Protein Preparation Wizard program of the MASTERO software package with the default parameters was used for the preparation of *Eh*PLK protein structure for docking. Before performing docking, bound ligand (PLP) was removed from the *Eh*PLK structure. This program was also used to determine the protonated state for histidine residues, highlights missing residues, add missing hydrogen atoms and remove water molecules beyond 5.0 Å of HET groups in the protein structure. Further energy minimization and optimization of *Eh*PLK structure was done using an OPLS2005 force field with a maximum root-mean-square deviation (RMSD) of 0.30 Å for atom displacement to terminate the minimization. In energy minimized *Eh*PLK structure, a receptor grid (inhibitor binding site) was created at the site of PLP binding. The receptor grid for the inhibitor covered all of the residues of the active site of PLP. A library of compounds was generated, specifically one that consisted of drugs from various databases, including the DrugBank, ZINC databases and various kinase inhibitor libraries (such as CDK2, CDK5, glycogen synthase kinase-3 (GSK3), AKT, casein kinase (CK2), tyrosine kinase Src and p38). Schrodinger’s Ligprep Wizard was used to generate library for each compound which generates up to 34 different conformations of each molecule for docking. Finally, docking of each compound in the *Eh*PLK receptor grid was carried out using the program GLIDE with the extra precision (XP) module. Nearly 28000 compounds were docked and listed according to the docking score. Based on the docking scores, availabilities and costs, we shortlisted and purchased best of five compounds, which were tested *in-vitro* and further on *E. histolytica* cultured cells. These compounds were obtained from ENAMINE Ltd., based in Ukraine and their chemical names are listed in **Table 1**.

**Table 1:** ZINC ID, IUPAC Names and docking scores of the chemical inhibitors docked at the PLP binding site of *Eh*PLK and human PLK (*Hs*PLK).

	ZINC ID	IUPAC Name	Glide Score	
			*Eh*PLK	*Hs*PLK
1	ZINC26710858	N-[4-(1,1-dioxo-1,2-thiazolidin-2-yl)phenyl]thieno[3,2-d]pyrimidin-4-amine	-8.93	-5.24
2	ZINC26710739	N-[4-(1,1-dioxo-1,2-thiazolidin-2-yl)phenyl]thieno[2,3-d]pyrimidin-4-amine	-9.40	-4.58
3	ZINC30519884	1-Ethyl-3-{[4-methoxy-3-(2-oxopyrrolidin-1-yl) phenyl]amino}pyrrolidine-2,5-dione	-8.80	-2.99
4	ZINC08346026	(4S)-6-ethyl-4-phenyl-1,3,4,7-tetrahydropyrrolo[3,4-d]pyrimidine-2,5-dione	-9.59	-4.80
5	ZINC6563174	(4S)-5-acetyl-6-methyl-4-phenyl-3,4-dihydro-1H-pyrimidin-2-one	-9.42	-6.47

Similarly, human PLK structural coordinates (PDB ID: 3KEU) were retrieved from Brookhaven Protein Data Bank RCSB-PDB (www.rcsb.org) and was prepared for the docking as done for the *Eh*PLK.

### *In-Vitro* Inhibition Assay

PLK kinetics was carried out as reported earlier (Tarique et al., 2020). Briefly, PLK enzymatic activity was determined in the absence of inhibitors for the reaction mixture containing *Eh*PLK (25 μg/ml (0.40 μM), 2 mM MgCl_2_ and 1 mM ATP, and activity was initiated with the addition of 20 μM of PL. The progress of reaction was determined on an Ultrospec 2100 pro spectrophotometer (GE Healthcare) which is proportional to an increase in absorbance at 388 nm due to formation of PLP (Di Salvo et al., 2004) whose concentration was measured using extinction coefficient of 5020 M^−1^cm^−1^ at 388 nm (Di Salvo et al., 2015). The five compounds were purchased and tested *in-vitro* for inhibition of *Eh*PLK activity. The inhibition kinetics assay was performed in reaction mixture (30 mM Na HEPES pH 7.5, 2 mM MgCl_2_, 1 mM ATP, 20 μM PL) with various concentrations (ranging from 10 µM to 250 µM) of the compound. Different concentration of the sub-stock of inhibitor was prepared in dimethyl sulfoxide (DMSO) (from the stock of 100 mM) such that constant volume of it was added to the reaction mixture to get the required final concentration in the reaction mixture. From the results, an IC_50_ value was calculated for those compounds which were showing inhibition of activity. Note that initially, the enzyme was incubated for three minutes with the compound in buffer containing 30 mM Na HEPES pH 7.5, 2 mM MgCl_2_ and 1 mM ATP. The reaction was started with the addition of pyridoxal to the reaction solution.

### *E. histolytica* Culture and Maintenance

The *E. histolytica* strain HM1:IMSS trophozoites were grown and maintained in a temperature and humidity controlled incubator set at 35.5°C. The cells were grown in TYI-S-33 medium supplemented with benzyl penicillin and streptomycin to avoid bacterial contamination. Incomplete TYI-S-33 medium was completed before trophozoite culture by adding Vitamin Mix (Sigma) and heat-inactivated adult bovine serum (Life technologies) to a final concentration of 1X and 15% respectively.

### Measurement of Cell Number

*E. histolytica* cell number was calculated using the hemocytometer. Trophozoites were harvested at different time intervals by centrifugation at 400 g for 5 minutes. Supernatant was disposed of and pellet was resuspended in new media. 10 μL of this cell suspension was put on a clean hemocytometer and covered with a cover slip. Cells were then counted in 4x4 square of the hemocytometer to ascertain cell number.

### Effects of Inhibitors on Growth Curve of *E. histolytica* Cultured Cells

Growth of trophozoites was measured for 72 hours. Five cell lines were created: 01-HM1, 02-Trophozoites + DMSO added only at time 0, 03-Trophozoites + DMSO added every 12 hours, 04-Trophozoites + inhibitor added only at time 0 and, 05-Trophozoites + inhibitor added every 12 hours. The inhibitor was added to a final concentration of 50 μM each time. Stock concentration of the both the inhibitors was 100 mM. The trophozoites were harvested in log phase by centrifuging the tube at 400 g for 5 min. In each case, old medium was discarded and the cells were resuspended in new medium. Cells were counted using a hemocytometer and each tube was inoculated with same number of trophozoites. The volume of media in a tube was 6 mL so to add 50 μM of inhibitor in each tube 3 μL of inhibitor from stock solution was added. At each time point, cells were harvested and cell number was determined using the method described above.

### Theoretical Prediction of ADME Parameters of Compounds

The compounds which showed enzyme inhibition were subjected to theoretical prediction of pharmacokinetic properties from their molecular structure using swissADME online server http://www.swissadme.ch/ (Daina et al., 2017). This server require SMILES of the compound and predicts ADME parameters (referred to Absorption, Distribution, Metabolism, Excretion/Elimination), pharmacokinetic properties, druglike nature and medicinal chemistry. It can also generates SMILES from the molecular structure of the compound.

## Results

### *Eh*PLK Enzyme Kinetics in the Presence of Inhibitors

**Figure 1:**
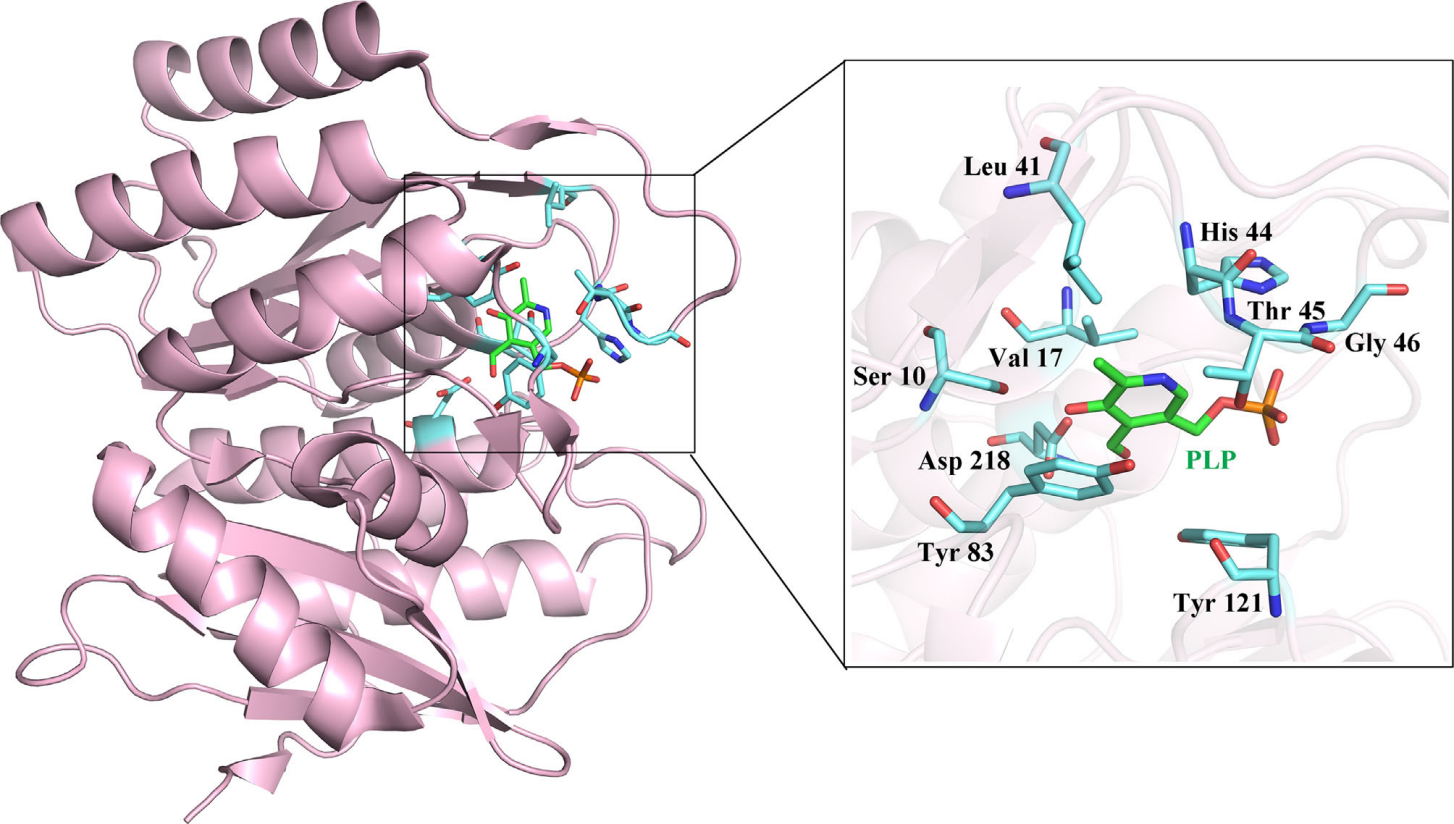
Structure of *Eh*PLK (PDB ID: 4S1I) (Tarique et al., 2020) with the bound pyridoxal 5`-phosphate (PLP) at the active site. Inset shows the enlarged view of various residues interacting with the PLP at the PLP binding site which is used for docking the inhibitors.

**Figure 2:**
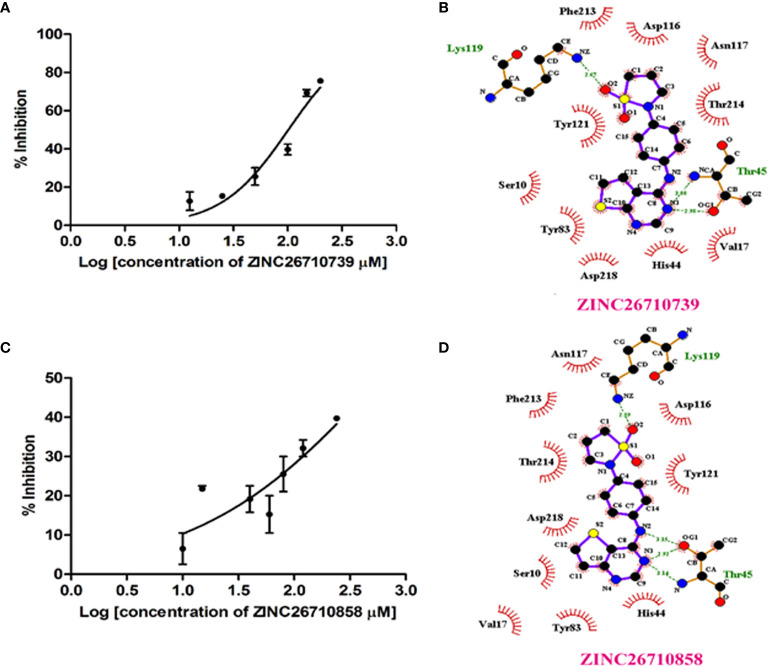
*Eh*PLK assays in the presence of inhibitors. **(A, C)** Results of inhibition kinetics assays performed with various concentrations of **(A)** ZINC26710739 (N-[4-(1,1-dioxo-1,2-thiazolidin-2-yl)phenyl]thieno[3,2-e]pyrimidin-4-amine and **(C)** ZINC26710858 (N-[4-(1,1-dioxo-1,2-thiazolidin-2-yl)phenyl]thieno[2,3-e]pyrimidin-4-amine) compound to determine the IC_50_ values. **(B, D)** LigPlot^+^ representations showing *Eh*PLK with the **(B)** docked ZINC26710739 and with **(D)** docked ZINC26710858 at the binding site for pyridoxal phosphate.

We purchased five compounds on the basis of how well they docked *in-silico* onto the *Eh*PLK structure (see docking scores in **Table 1**) at the PLP binding site (**Figure 1**) (Tarique et al., 2020) and their availability, to check their effects as inhibitors on the *Eh*PLK enzyme. Amino acid residues at PLP binding site in *Eh*PLK are Ser10, Val17, Leu41, His44, Thr45, Gly46, Tyr83, Tyr121 and Asp218. All the 5 compounds docked very well at the PLP binding site. In the presence of each compound, the rate of the conversion of PL to PLP was monitored. Out of these five tested compounds, two of them (ZINC26710739 and ZINC26710858) showed inhibition with IC_50_ values approximately 100 and 250 µM respectively (**Figure 2**). However, these compounds did not show complete inhibition of activity upon further increasing the concentration. The maximum inhibition showed by these two compound ranges from 50-70% of maximum PLK activity. Both of them interacting with most of the residues of PLP binding site of *Eh*PLK (**Figure 2**). Thus, these compounds showing inhibition in the activity probably by competing with the PL for the binding site.

**Figure 3:**
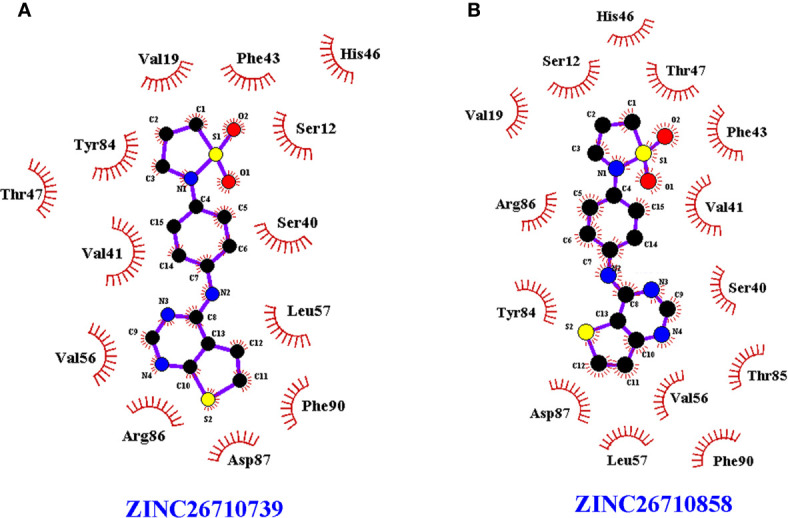
LigPlot^+^ of human PLK docked with the compounds. LigPlot^+^ showing human PLK docked with the **(A)** ZINC26710739 and **(B)** ZINC26710858 at the binding site for pyridoxal phosphate which shows that residues of human PLK interacting with these compounds are different compared to *Eh*PLK structure.

However, both of the compounds showed lower docking scores (**Table 1**) when docked at the PLP binding site of human PLK compared to *Eh*PLK, indicating them to be more selective in inhibiting *Eh*PLK than human PLK, at least *in-silico* (**Figure 3**).

### Effects of Inhibitors on *E. histolytica* Cultured Cells

**Figure 4:**
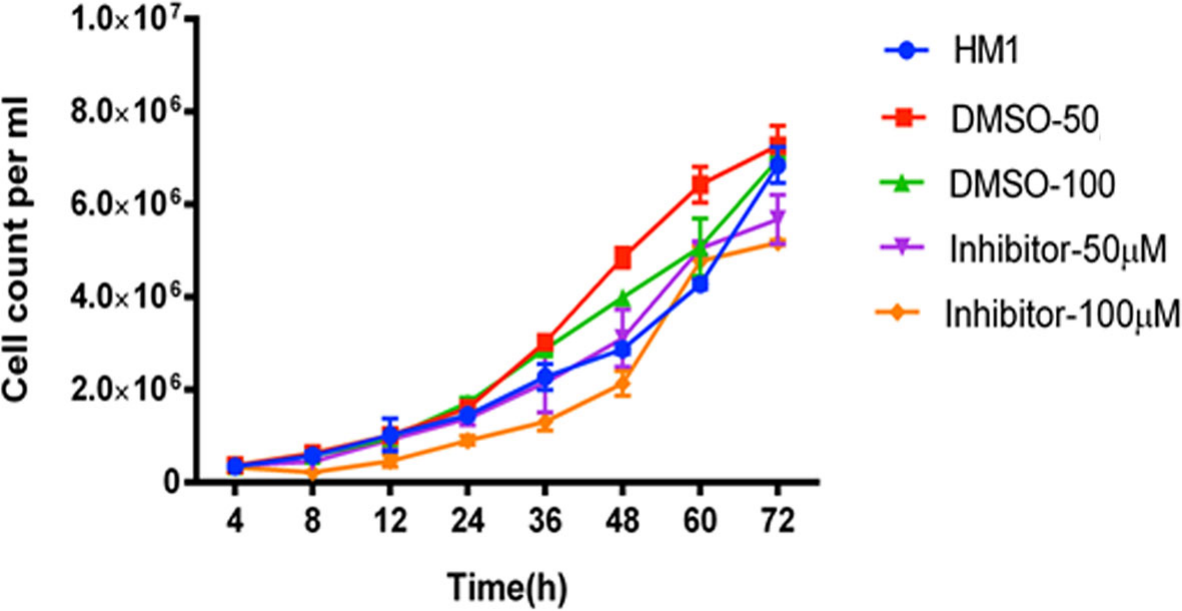
Growth kinetics of cells of the HM1 cell line with or without inhibitor (ZINC26710739) at two different concentrations, 50 μM and 100 μM, over a period of 72 hours. Equal number of cells (3x10^5^ mL^-1^) were inoculated into each fresh growth medium. The cells were harvested at indicated time points, resuspended in PBS and counted using a hemocytometer. Data are reported as mean ± SD of two independent experiments. HM1- Wild type trophozoites, DMSO-50 - Trophozoites + 3 μL DMSO, DMSO-100- Trophozoites + 6 µL DMSO, Inhibitor- 50 μM Trophozoites + 3 μL Inhibitor ZINC26710739 (50 μM), Inhibitor- 100 μM- Trophozoites + 6 μL Inhibitor ZINC26710739 (100 μM). Growth of the HM1 cell line was compromised for the 12 hours but regained after it in the presence of inhibitor.

**Figure 5:**
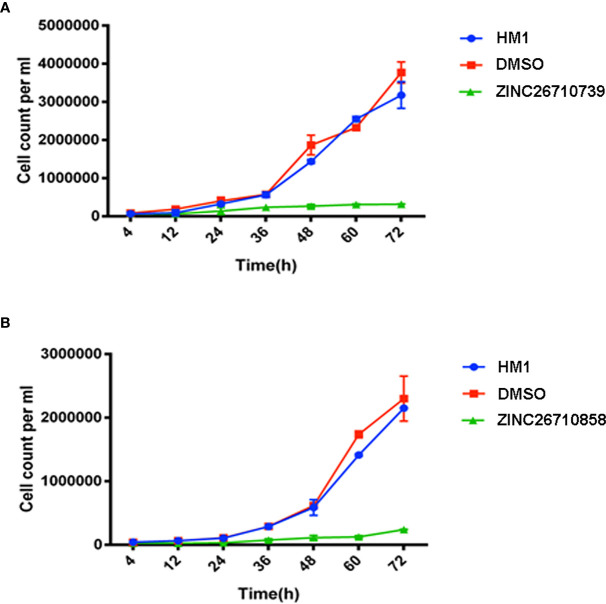
Growth kinetics of HM1 cells with or without inhibitors **(A)** ZINC26710739 and **(B)** ZINC26710858 each at a concentration of 50 μM (added after every 12 hours) over a period of 72 hours. Equal number of cells, specifically 6x10^4^ mL^-1^ and 3.5x10^4^ mL^-1^, respectively, were inoculated into fresh growth medium. The cells were harvested at the indicated time points, resuspended in PBS and counted using a hemocytometer. Data are reported as means ± SD of three independent experiments. HM1- Wild type trophozoites, DMSO- Trophozoites + DMSO, ZINC26710739- Trophozoites + 3 μL Inhibitor ZINC26710739 (50 μM), ZINC26710858- Trophozoites + 3 μL Inhibitor ZINC26710858 (50 μM).

To elucidate the effects of these two compounds on *E. histolytica* strain HM1:IMSS, we added each of the tested compounds into different samples of growing trophozoites and studied the growth kinetics for 72 hours. The trophozoites to which 50 μM and 100 μM concentrations of ZINC26710739 inhibitor were added, initially showed slow and inhibitory growth levels, but regained their normal growth after 12 hours (**Figure 4**), suggesting that either the drug was metabolized or removed from the cells or in some other way disabled within this time period. Therefore, we decided to repeat administration of the dose every 12 hours: in this case, trophozoite growth was much slower and was inhibited by up to 91.5% over time (**Figure 5A**). These results indicated that this inhibitor is indeed lethal for *Entamoeba* growth but a repeated dose is required to see the maximum effect. Inhibition studies for ZINC26710858 were also conducted in a similar fashion, and this inhibitor also showed a detrimental effect on *Entamoeba* growth, as the growth was inhibited by 89.5% (**Figure 5B**). All of the comparisons were made against HM1:IMSS trophozoites added with same volume of DMSO every 12 hours. Various literatures are available where the standard drugs used for treatment of Amoebiasis are Metronidazole and tinidazole and their IC_50_ value was calculated in cultured cells. The IC_50_ of metronidazole came out to be in range from 5-10 μM (Bansal et al., 2004; Jarrad et al., 2016). Our compounds inhibited amoebic growth by 90% in cultured cells at a concentration of 50 μM. In a study by Bansal et al., 2004; IC_50_ for Metronidazole was calculated to be 9.5 μM but 90% inhibition in the growth was observed at a concentration of around 60 μM, which is comparable to the present study.

These studies also corroborated our findings that this enzyme is indeed very important for the normal growth and homeostasis of *Entamoeba* trophozoites and that inhibiting this enzyme leads to death of the trophozoites.

### Theoretical Prediction of ADME Parameters of the Compounds

Both the compounds which showed inhibition were subjected to theoretical prediction of ADME parameters and found that both follows ADME and Lipinski’s rule (**Table S1**). Both of them showed high gastrointestinal absorption and do not show blood brain barrier permeation, indicating it to be lead compound for the further drug development.

## Discussion

PLP is one of the most important cofactors required for the proper functioning of various enzymes. Enzyme variants lacking PLP show inability to perform its function (Bonner et al., 2005), indicating necessity of cofactor PLP. Pyridoxal kinase is the enzymes which synthesize PLP in *E. histolytica*. Based on the previous studies that had shown *Eh*PLK to be essential for the growth and survival of this pathogen. Besides this, *Eh*PLK is structurally different from its human counterpart and also in phylogenetic tree, *Eh*PLK protein is far from the human PLK indicating it to be different at the sequence level as well (Tarique et al., 2020). In the present study we have performed *in-silico* inhibitor screening against this enzyme and identified two small molecules: ZINC26710739 and ZINC26710858 that showed inhibition of *Eh*PLK in our kinetics studies. The potential effectiveness of these inhibitors was tested on cultured *E. histolytica* cells as well, which showed promising results in inhibiting the growth of *E. histolytica*. The IC_50_ of these inhibitors were measured to be between 100 and 250 μM. These suboptimal levels also helped explain why boosters of these inhibitors were required to show relatively sustained detrimental effects on the growth of this protozoan parasite. These compounds seem to be competing with the pyridoxal for the pyridoxal binding site. These chemical inhibitors appear to be potent lead molecules, and chemical modification might increase their effectiveness tremendously.

Pyridoxal-amino acid adducts like PT5 and PT3 have shown promising results in inhibiting PLK in *Plasmodium falciparum*, it nonetheless also, to a moderate extent, affected the host too (Müller et al., 2009). Similarly, 4-deoxypyridoxine, a vitamin B_6_ antagonist meant to treat sleeping sickness was shown to be effective on porcine cell lines not on *Trypanosoma brucei* (Ferguson et al., 2005). Other PLP-mimicking compounds such as roscovitine, theophylline and gingotoxins were shown to inhibit PLKs but also displayed neurotoxicity and cross-reactivity with host protein kinases such as CDK2 and CDK5, indicating the unsuitability of these compounds for human consumption (Tang et al., 2005; Gandhi et al., 2012). The promiscuous nature of the PLK active site has thus made designing a potent species-specific inhibitor against PLK quite challenging. Co-crystallizations of vitamin B_6_ inhibitors with host protein kinases and PLK should yield information allowing us to design two different classes of inhibitors, the first one specifically targeting the host protein and the second one specifically targeting the pathogen PLK.

## Conclusions

*Eh*PLK is a promising drug target which is critical for the survival of *E. histolytica.* Inhibitors tested against *Eh*PLK, namely ZINC26710739 and ZINC26710858, have shown encouraging results in inhibiting the growth of *E. histolytica* cultured cells. *In-silico* docking of these inhibitors with the human PLK showed lower docking score compared to *Eh*PLK prompting us to believe that these drug molecules may have lethal effect on *E. histolytica* in comparison to host. The overall results presented in this study have shown that *Eh*PLK is a drug target and the above lead molecules can be exploited for drug discovery against this protozoan parasite. The overall work contributes to the ongoing efforts for targeting pyridoxal kinases in other protozoan parasites.

## Data Availability Statement

The raw data supporting the conclusions of this article will be made available by the authors, without undue reservation.

## Author Contributions

Conceived the idea and designed the experiments: SD, FT, PT, and SG. Inhibition assays and inhibitor screening: SD and FT. *In vivo* experiments: PT. Writing-original draft manuscript: SD, FT, and PT. Formal analysis, funding acquisition, methodology, software, supervision, and validation: SG. All authors contributed to the article and approved the submitted version.

## Funding

This project is funded by the Department of Biotechnology [BT/PR27229/MED/29/1248/2017], Government of India, New Delhi.

## Conflict of Interest

The authors declare that the research was conducted in the absence of any commercial or financial relationships that could be construed as a potential conflict of interest.
