# Supplementary material for: Inhibiting Pyridoxal Kinase of Entamoeba histolytica Is Lethal for This Pathogen
Source: Front Cell Infect Microbiol. 2021 Apr 16;11:660466. doi: 10.3389/fcimb.2021.660466 (PMC8085340; doi:10.3389/fcimb.2021.660466)
Supplement: Supplementary file 1 [file Table_1.docx]

*Supporting information*

Inhibiting pyridoxal kinase of *Entamoeba histolytica* is lethal for this pathogen

Suneeta Devi^1^, Priya Tomar^1^, Faisal Tarique Khaja^1,2^, and Samudrala Gourinath^1^*

^1^Structural Biology Laboratory, School of Life Sciences, Jawaharlal Nehru University, New Delhi, India

^2^Department of Molecular Reproduction, Development and Genetics, Indian Institute of Science, Bangalore, India

*To whom correspondence should be addressed:

**Professor Samudrala Gourinath**

School of Life Sciences

Jawaharlal Nehru University

New Delhi-110067

India.

E-mail: sgourinath@mail.jnu.ac.in

Tel: +91 11 2670 4513

Fax: +91 11 11 2674 2558

|  |  | ZINC26710858 | ZINC26710739 | ZINC30519884 | ZINC08346026 | ZINC6563174 |
| --- | --- | --- | --- | --- | --- | --- |
| 1. | Molecular Weight | 346.43 | 346.43 | 331.37 | 257.29 | 230.26 |
| 2. | Number of rotatable bonds | 3 | 3 | 5 | 2 | 2 |
| 3. | Number of H-bond acceptors | 4 | 4 | 4 | 2 | 2 |
| 4. | Number of H-bond donors | 1 | 1 | 1 | 2 | 2 |
| 5. | GI absorption | High | High | High | High | High |
| 6. | BBB permeant | No | No | No | No | Yes |
| 7. | Lipinski violations | 0 | 0 | 0 | 0 | 0 |
| 8. | Lead likeness  violations | 0 | 0 | 0 | 0 | 1 |
| 9. | Synthetic accessibility  (from 1 (very easy) to 10 (very difficult) | 3.21 | 3.13 | 3.09 | 3.61 | 3.34 |

**Table S1.** Theoretical prediction of ADME parameters of the compounds.
